# The Involvement of Human Papilloma Virus in Gastrointestinal Cancers

**DOI:** 10.3390/cancers14112607

**Published:** 2022-05-25

**Authors:** Jacek Baj, Alicja Forma, Iga Dudek, Zuzanna Chilimoniuk, Maciej Dobosz, Michał Dobrzyński, Grzegorz Teresiński, Grzegorz Buszewicz, Jolanta Flieger, Piero Portincasa

**Affiliations:** 1 Department of Anatomy, Medical University of Lublin, Jaczewskiego 4, 20-090 Lublin, Poland; 2 Department of Forensic Medicine, Medical University of Lublin, Jaczewskiego 8b, 20-090 Lublin, Poland; iga.dudek6@gmail.com (I.D.); zuzia.chil@gmail.com (Z.C.); macdob.98@gmail.com (M.D.); mdobrzyski4@gmail.com (M.D.); grzegorz.teresinski@umlub.pl (G.T.); g.buszewicz@umlub.pl (G.B.); 3 Department of Analytical Chemistry, Medical University of Lublin, Chodźki 4A, 20-093 Lublin, Poland; j.flieger@umlub.pl; 4 Clinica Medica “A. Murri”, Department of Biomedical Sciences & Human Oncology, University of Bari Medical School, 70124 Bari, Italy; piero.portincasa@uniba.it

**Keywords:** human papilloma virus, gastrointestinal cancer, esophageal cancer, liver cancer, gastric cancer, colorectal cancer, anal cancer, carcinogenesis

## Abstract

**Simple Summary:**

Mounting evidence suggests a relationship between *Human Papilloma Virus* (HPV) infection and the occurrence of neoplastic transformations within oral, pharyngeal, and anal cancers. Other segments of the intestinal tract can also be involved. Knowledge about the association between HPV infection and gastrointestinal carcinogenesis is crucial for both cancer prevention and patient care. Unfortunately, definite conclusions cannot be drawn yet, due to the high number of contradictions in the published papers.

**Abstract:**

Human Papilloma Virus (HPV) is one of the most common sexually transmitted infections worldwide. HPV infection has a strong relationship with the onset of cervix uteri, vagina, penis, anus, and oropharynx, but also tonsils and tongue cancers. Some epidemiological data indicate that except for gynecologic cancers, HPV infection can be one of the risk factors associated with a greater risk of induction and progression of gastrointestinal cancers. Data, however, remain contradictory and definite conclusions cannot be drawn, so far. The following review aims to organize recent evidence and summarize the current state of knowledge regarding the association between HPV infection and gastrointestinal tumors primarily focusing on esophageal, liver, gastric, colorectal, and anal cancers.

## 1. Introduction

**Figure 1:**
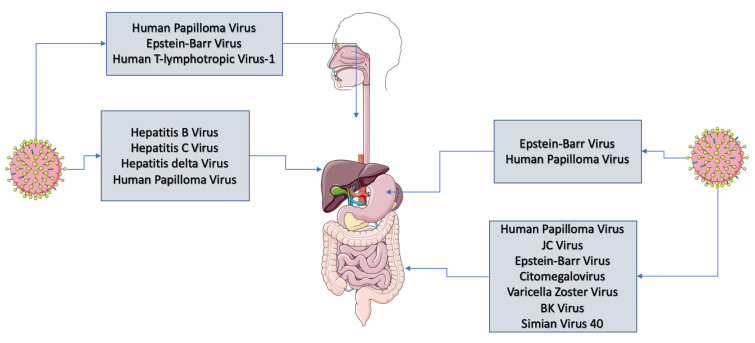
Viruses that might induce carcinogenesis within the gastrointestinal tract.

*Human Papilloma Virus* (HPV) belongs to the group of DNA viruses and Papovaviridae family, constituting a group of 174 characterized and documented types with the newest species being constantly discovered [1]. This virus reveals the high tropism rate mainly in the epithelia of the upper respiratory tract, genitals, as well as skin. The HPV family is divided into two groups according to their oncogenic potential—low-risk and high-risk HPV, which are either responsible for benign malignancies or major cancers correspondingly [2]. According to the International Agency for Research on Cancer (IARC), several high-risk HPV have been listed, namely 16, 18, 31, 33, 35, 39, 45, 51, 52, 56, 58, and 59, and these types are mainly responsible for carcinogenesis [3]. On the other hand, low-risk HPV such as type 6, 11, 42, 43, and 44 have an impact on benign hyperproliferative lesions or papillomatosis [4]. The main way of transmission of HPV includes sexual contacts and the most commonly infected areas include the genitals, anus, mouth, or throat. In addition to sexual intercourse, HPV transmission is facilitated by smoking, multiple partners, deficiency of some vitamins such as vitamin A, as well as the defective immune system [5]. It is estimated that, worldwide, there are more than 550,000 new patients annually who suffer from various malignancies caused by the infection of HPV [6]. Except for common genital cancers, esophageal squamous cell carcinoma (ESCC), colorectal cancers, conjunctiva carcinoma along with oropharyngeal cancers, HPV is considered to play a role in the development of neoplastic transformations within the gastric mucosa, which may eventually lead to the progression to gastric cancer [7]. Gastric cancer is the third cause of death associated with cancer occurrence among both males and females (approximately 723,000 cases of death per year). Such estimates are more commonly observed in men in comparison to women with the highest rates in East Asia, East Europe, and South Africa [8,9]. Approximately, 75% of gastric cancer cases are associated with the infection of *Helicobacter pylori* (*H. pylori*) [10,11,12]. Likewise, other viral infections such as *Epstein–Barr Virus* (EBV), for instance, might be crucial in the development of this malignancy [13] (Figure 1).

In the following paragraphs, we focus on the malignancies of the gastrointestinal tract that could be induced by the HPV infection but are not as common in the clinical practice as HPV-triggered gynecological cancers, primarily considering esophageal cancer, liver cancer, gastric cancer, colorectal cancer, and anal cancer.

## 2. Human Papillomavirus Description

*Human Papilloma Virus* (HPV) is representative of the Papillomaviridiae family having a double-stranded circular DNA genome with a virion size of approximately 55 nm in diameter [14]. To date, over 200 types of HPV have been described, and the number of further types discovered continues to rise [15]. The individual types can be further classified into five large genera—alfa-papillomas, beta-papillomas, gamma-papillomas, mu-papillomas, and nu-papillomas. The classification into genera is based on the ORF (open reading frame) nucleotide sequence encoding the L1 capsid protein, with each type of HPV sharing less than 60% similarity within the L1 genome. Among the genera, the individual HPV types are numerically classified according to the Papillomavirus Workshops held in 1995 and the division is based on less than 90% similarity in the L1 protein-coding sequence [16]. Alpha-papillomas have an affinity for mucous membranes and skin, while the other types affect only skin [17]. Mucosal types are further subdivided into high-risk and low-risk groups that do not typically cause neoplasia [18]. HPV is the most common sexually transmitted infection (STI) and the leading cause of cervical cancer in women. Moreover, the virus causes vulvar, vaginal, penile, anal, oropharyngeal, and cutaneous carcinomas. Low-risk HPV types are associated with benign skin lesions such as genital, flat, or common warts [19]. Usually, except for papillomas, such lesions resolve with time and are cleared by the immune response. Regarding high-risk HPV types, 12 of them have been classified as group 1 carcinogens to humans—type 16, 18, 31, 33, 35, 39, 45, 51, 52, 56, 58, and 59 according to IARC [20]. In 2018, the total number of cancers attributable to HPV infection accounted for 690,000 cases worldwide with an age-standardized incidence rate (ASIR) of 8.0 cases per 100,000 person-years [21]. Of these cases, about 80% were cervical cancers (n = 570,000). Remaining carcinomas, counted as new cases attributable to the disease, occurred with the frequency in the presented order: oropharyngeal carcinoma (n = 42,000), anus squamous cell carcinoma (n = 29,000), penis carcinoma (n = 18,000), vagina carcinoma (n = 14,000), vulva carcinoma (n = 11,000), oral cavity cancer (n = 5900), and larynx cancer (n = 4100). According to GLOBOCAN statistics from 2020, cervical cancer represents the 9th cancer worldwide in terms of incidence, while among women, it is in the 4th position both in terms of incidence (n = 604,127) and as the leading cause of cancer death (n = 341,831) [22]. Across all continents, invasive cervical cancer is most often caused by persistent HPV-16 infection, followed by HPV-18 [23]. HPV-16 is also the main type responsible for oropharyngeal squamous cell carcinomas [24]. Overall, HPV-18 and 16 account for 72% and HPV 31, 33, 45, 52, and 58 for an additional 17% of all HPV-attributable cancer cases [21]. Most anogenital HPV infections are acquired through sexual contact and the possibility of infection increases with the number of sexual partners [25]. HPV infections are very common in young women with a peak around 20–25 years of age. Later, the prevalence of infection remains on the stable level of 5–10%. In total, approximately 80% of sexually active women contract an HPV infection during their lifetime and, in most cases, it resolves within 6 to 12 months [26]. Viral transmission is believed to be higher in female-male rather than male-female contacts [27,28]. The prevalence of genital HPV infection is similar in men and women [29]. Among men, regardless of their younger or older age, the infection rate remains on a stable level and varies very little, contrary to women. The highest risk of anal HPV infection is seen in homosexual and HIV-infected men. HPV infection can also be acquired through skin abrasions, by kissing and digital contact, with finger–genital contact, perinatal transition, and oral sex [30]. Concerning perinatal horizontal transmission from mother to child, there is evidence that caesarean section can reduce the incidence of HPV perinatal infection by approximately 46% [31]. Interestingly, human papillomavirus can be detected in female virgins with the prevalence varying from 0 to 51.1%, and some studies commonly found HPV on the surface of medical instruments and public environments [32].

Concerning gastrointestinal tract (GIT) cancers, they accounted for 26% of new cancer cases worldwide and 35% of all cancer-related deaths in 2018, with the highest prevalence of colon cancer (1.8 million new cases) [33]. Based on predictions, by 2040, the global numbers of new cases and deaths caused by GIT cancers will increase, respectively, by 58% and 78%. HPV infection is a controversial risk factor among esophageal, gastric, liver, and colorectal malignancies with many conflicting studies, which is presented further in the review.

## 3. HPV Oncogenesis

The HPV genome consists of three major functional regions including early, late, and long control regions, which are separated by early (pA_E_) and late polyadenylation sites (pA_L_) [14]. The early region encodes six common open reading frames (E1, E2, E4, E5, E6, and E7) translating individual proteins, whereas the late region encodes L1 and L2 ORF for translation of a major (L1) and minor (L2) capsid protein. The long control region (LCR) contains transcription factor binding sites and the origin of replication, without a protein-coding function as with the rest of the regions. HPV infection initiates with the interaction of the L1 capsid protein on the viral capsid with the heparan sulfonated proteoglycan found on the cytoplastic membrane of the cell at the basal layer of the epithelium [26,34]. The life cycle of papillomavirus is strictly related to keratinocytes, which are present in the epidermis of the skin and stratified squamous epithelia of the genitals, oral cavity, and esophagus—new virions can only be assembled in keratinocytes undergoing terminal differentiation [35]. HPV gains access to basal epithelial cells by epithelial trauma and maintains the viral episome at a low copy number in the infected cells [36]. It is believed that for the persistent lesion to develop, papillomavirus must infect long-lived epithelial stem or stem-like cells and that the local microenvironment and growth factors released around the wound-healing area may play a crucial role for virus reservoir [37]. After the entry, HPV migrates to the host cell nucleus and rapidly increases its viral DNA genome by replication, dependent on the viral E1 and E2 replicative proteins [38]. Low-copy episomes are established in undifferentiated cells and replicated viral genomes are distributed equally to two daughter cells, of which one migrates toward the suprabasal layer and undergoes differentiation. Upon differentiation, thousands of genome copies are produced, and finally, among terminally differentiated cells of the epithelium, the L1 and L2 capsid proteins are expressed, and viral particles are released with the most exterior part of the squamous cells layer.

In the 1980s, the first reports of the possibility of HPV-induced cell transformation appeared and the greatest importance was assigned to the E7 gene encoding a protein of the same name [39,40,41,42,43,44]. Subsequent in vitro studies conducted on human keratinocytes showed that the E7 protein closely cooperates with the E6 protein and only their expression together leads to the cell immortalization [45,46,47]. It is claimed that the E7 protein supports further DNA synthesis among cells of the suprabasal layer of the epithelium, which is normally limited only to the basal cells [48]. The most frequently described E7 protein feature responsible for oncogenesis is binding to the pRb family of proteins pRb, p107, and 130 [49,50,51]. pRb has an ability to interact with EF2 transcription factors involved in the activation of DNA replication and the regulation of the G1-to-S phase transition [52]. In a normal manner, pRb is active in a hypophosphorylated form and prevents S-phase entry [53]. It represses transcription in three distinctive ways—by binding to the E2F activation site, blocking the assembly of pre-initiation complexes, and associating with complexes that modify the chromatin structure [54]. The E7 protein-mediated pRb destruction leads to the release of EF2 factors and activation of genes responsible for cell proliferation [55]. An additional mechanism through which E7 can be responsible for oncogenic transformation is pRb degradation via a ubiquitin-proteasome pathway [56,57]. In addition to the well-known interaction with pRb, the E7 protein has been shown to contribute to genomic instability [58,59]. HPV-16 E7 induces abnormal centrosome duplication and aberrant mitotic spindle formation. Interestingly, the genomic instability is present at a very early stage of high-risk HPV infection [58]. E7 induces centriole multiplication via the up-regulation of Polo-like kinase 4 in HPV-16-expressing cells [60] and alters the recruitment of gamma-tubulin to the centrosome [61]. In addition, E7 can delocalize dynein from mitotic spindles and interact with NuMA (nuclear mitotic apparatus protein 1), resulting in mitotic disruptions [62].

In addition to well-known E7 protein-transforming properties, it was quickly noticed that similar characteristics were exerted by the E6 protein encoded by different types of human papillomaviruses. E6 can bind to the p53 protein, which, in turn, correlates with in vitro-transforming activity and in vivo clinical behavior [63]. The degradation of the p53 protein is ATP-dependent and involves the ubiquitin-dependent protease system [64]. Binding to the p53 is mediated by E6-AP (associating protein) [65,66,67]. In vitro studies showed that the downregulation of E6-AP expression on HPV-positive cells leads to growth suppression and p53 accumulation [68] and that the stability of the E6 protein is strictly dependent upon E6-AP presence [69]. One study found a complete loss of the oncogenic potential in mice nulligenic for the E6-associated protein [70]. Notably, the degradation properties of E6 are not limited only to E6-AP binding [71], and this association alone is not sufficient [72]. Another property of the E6 oncogenic protein is the ability to activate telomerase, the enzyme responsible for the synthesis of telomere repeat sequences, which prolongs the life of HPV-16-infected cells and is independent of p53 degradation [73]. The telomeres are the complexes consisting of tandem repeat DNA sequences found at the end of eukaryotic chromosomes, and they function as a molecular clock that controls the replicative capacity of human cells [74]. The activation of telomerase is mediated by E6-enhanced expression of the human telomerase reverse transcriptase (hTERT) catalytic subunit. The binding of E6 to myc proteins that are closely related to hTERT activation is believed to play a role [75]. Moreover, other targets for the E6 protein are two isoforms of NFX-1-NFX1-123 and NFX1-91. NFX1-91 is a repressor of telomerase and, by binding with E6, it undergoes ubiquitination and degradation [76]. On the other hand, NFX1-123 interacts with HPV type 16 E6 and increases hTERT mRNA levels [77] (Table 1). Morgan et al. demonstrated that the HPV E6 protein stimulates the proto-oncogenic transcription factor STAT3 and the process is mediated by the IL-6 and a subsequent activation of the transcription factor NFκB [78]. The authors indicate that the activation of the whole axis remains crucial in the further proliferation and survival of cancer cells. Other studies indicate that HPV E7 is involved in the activation of the STAT-5 phosphorylation promoting further HPV replication through activating the ATM DNA damage response [79]. Further, an increased expression of EGFR and a subsequent activation of JNK/c-Jun signaling are crucial for further activation of HPV E6 and E7 proteins [80]. The Hippo signaling pathway combined with the EGFR signaling interact with HPV E6 and E7 oncoproteins, enhancing tumor growth, proliferation, and migration of the cancerous cells [81,82]. Generally, HPV oncoproteins—primarily E5, E6, and E7—are involved in facilitating numerous signaling pathways, providing an environment beneficial for the viral replication and further oncogenesis [83].

Apoptosis, as a process of programmed cell death, is one of the most important cellular processes that regulates the quantity of cells. Several tumors can inhibit apoptosis, thereby preventing cell death and allowing the cells to multiply uncontrollably. The E6 and E7 oncoproteins produced by HPV interfere with apoptotic processes. The E6 protein leads to the degradation of the pro-apoptotic Bak protein [87,88]. It also inhibits the action of the Bax protein and downregulates the expression of Bax mRNA, which interacts with the Bak protein in the process of controlled cell death [89,90]. Moreover, the E6 protein can bind directly to the Fas-associated Death Domain (FADD), preventing Fas-induced apoptosis [86]. Both E6 and E7 proteins attenuate the action of transforming growth factor- β2 (TGF-β2) in keratinocytes, which is characterized by regulation of the cell cycle and tissue remodeling [112].

Another mechanism responsible for the neoplastic transformation of many human tissues is related to the downregulation of miRNA, non-coding small RNAs that are posttranscriptional mRNA regulators [91]. In one study, after sequencing RNAs of human foreskin keratinocytes expressing either HPV16 E6/E7 proteins or control vectors, 51 differentially expressed miRNAs turned out to be associated with the modulation of 1456 target mRNAs [92]. Another similar research showed a dysregulation of 60 and 90 miRNAs in the presence of either the E6 or E7 protein, respectively; further, the joint expression of both proteins was associated with the alterations in the levels of 64 miRNAs [93]. For example, HPV proteins alter the miR-203 expression, miRNA that is mostly present in suprabasal layers of stratified epithelia and is responsible for suppressing the proliferative capacity of epithelial cells upon differentiation [113]. The HPV E6 oncoprotein can increase the level of miR-20b inducing morphological cell alterations in cervical carcinoma tissue [94].

## 4. HPV Detection Methods

Concerning human papillomavirus detection, the determination of anti-HPV antibodies is of limited diagnostic value as they may persist for many years and their presence does not distinguish between past and present infection [114,115]. In addition, human papillomavirus cannot be grown in conventional cell cultures. Thus, detecting viral nucleic acid is of the greatest importance. Most methods of detecting HPV infection are reserved for prompt diagnosis and early prevention of the development of cervical cancer in women. For this purpose, many tests have been developed to investigate the presence of HPV-DNA in cervical specimens, and five of them have been approved by the Food and Drugs Administration for wider use—Hybrid Capture II HPV DNA test, Cervista HPV HR, Cervista HPV 16/18, Cobas HPV test, and APTIMA HPV Assay [116]. One of the methods of HPV-DNA detection is in situ hybridization (ISH), which relies on the usage of labeled probes that specifically hybridize to DNA, but the disadvantage of ISH is its limited specificity [114,117]. The undoubted advantage of this method, however, is the determination of the exact location of HPV in the sample—the virus integrated with the genetic material reveals itself as a punctuated signal, while the episomal form reveals itself as a diffused signal [118]. Other techniques that use hybridization are Southern blot, dot blot, and reverse blot and non-radioactive hybrid capture [119]. The most sensitive method is the polymerase chain reaction (PCR), which can detect a single genetic material per 100,000 cells while the previously discussed ISH method provides a detection of one DNA per 100 cells [120]. PCR is the reaction relying on the set of oligonucleotide primers and thermostable DNA polymerase that will elongate the genetic material [121]. The process depends on DNA denaturation followed by the annealing of primers and DNA replication. There are multiple PCR methods using consensus primers that target mainly conserved regions of L1 ORF or type-specific primers, allowing the detection of individual HPV types [121]. It should be noted that PCR must be performed on fresh or frozen tissue [122,123]. Doeberitz in his work proposed three criteria that should be met to accept HPV as the direct cause of malignancy: (1) for each cancer cell, there should be at least one HPV genome, (2) cells should transcribe and translate oncoproteins E6 and E7, and (3) there should be the loss of tumorigenic ability with the inhibition of E6/E7 activity [124,125].

## 5. HPV and Esophageal Cancer

Esophageal cancer is currently the 10th most commonly diagnosed cancer worldwide, with an estimated number of 604,100 cases, being, at the same time, the 6th most common cause of cancer-related deaths with an estimated number of 544,076 deaths [22]. Esophageal cancer constitutes a malignancy that is usually about three to four times more prevalent in males rather than in females. Two major types of a cancer can be distinguished—(ESCC) and esophageal adenocarcinoma (EAC) [126]. For ESCC, the main risk factors primarily include increasing age, male sex, cigarette smoking, and alcohol consumption, while gender, cigarette smoking, gastroesophageal reflux disease (leading to Barret’s dysplasia (BD)), and obesity constitute the major risk factors for EAC [127,128]. The involvement of human papillomavirus (HPV) in the onset of ESCC remains controversial—positive relationships between those two factors have already been reported in many Chinese studies, whereas studies from the Western countries usually report no clear associations [129,130,131,132]. One explanation for this might be that HPV DNA contamination cannot be ruled out as a cause for high HPV prevalence in ESCC tissue [131]. Rajendra et al. proved a strong association between high-risk HPV and BD, as well as EAC. Amongst 261 patients, 81 were positive for HPV DNA. HPV was mostly detected at the transformation zone in both controls and BE. Compared with controls (18.0%), HPV positivity was significantly more common in BD (68.6%, incidence rate ratio (IRR) 2.94, 95% confidence interval (CI) 1.78–4.85, *p* < 0.001) and EAC (66.7%, IRR 2.87, 95% CI 1.69–4.86, *p* < 0.001) [133]. Based on the results of the study, they conducted a further investigation on whether HPV-positive and HPV-negative EAC demonstrate the distinct genomic difference. The HPV-positive cohort harbored approximately 50% fewer non-silent somatic mutations compared to the virus-negative patients with esophageal cancer (1.31 mutations/Mb vs. 2.56 mutations/Mb, *p* = 0.048). Regarding the TP53 aberrations, in the HPV-positive EAC group, they were absent, whilst 50% of the HPV-negative patients with EAC tended to exhibit the TP53 mutations. The results indicate different biological mechanisms of tumor formation regarding HPV-positive and HPV-negative EAC [134]. In the study conducted by Agalliu et al., HPV16 and other oral alpha, beta, and gamma HPVs are not associated with the risk of esophageal cancer [135]. The relationship between ESCC and HPV still requires further validation, although the strong association between EAC and high risk-HPVs has been proved. A meta-analysis considering the relationship between HPV infection and overall esophageal cancer survival has been conducted. It indicated that HPV infection may not be of prognostic utility in the evaluation of factors contributing to esophageal cancer [136]. Esophageal cancer treatment consists of two main parts—neoadjuvant concurrent chemoradiotherapy (CCRT) and surgery [137]. Randomized trials have demonstrated a convincing survival rate benefit through the use of neoadjuvant CCRT followed by a surgery for the patients with locally advanced esophageal cancer [138]. Bognar et al. found an association between the HPV infection and a poor response to oncological treatment, as well as a decreased overall survival, proving to be a negative prognostic factor in patients with ESCC [139]. Therefore, so far, the relationship between the HPV infection and esophageal cancer cannot be clearly established. Results of the research mentioned above show that there might be a connection between HPV prevalence and EA occurrence; however, there seems to be a lack of reliable data considering the HPV impact on ESCC. A further investigation needs to be conducted.

## 6. HPV and Primary Liver Cancer

Primary liver cancer constitutes the 6th most prevalent cancer, as well as the 3rd leading cause of cancer death worldwide in 2020, with approximately 906,000 new cases and 830,000 deaths reported. Primary liver cancer includes hepatocellular carcinoma (HCC) (reported as about 75–85% of the total number of cases) and intrahepatic cholangiocarcinoma (10–15%); other liver cancers constitute those of rare types [22,140]. Chronic infection with hepatitis B virus (HBV) or hepatitis C virus (HCV), aflatoxin-contaminated foods, and heavy alcohol intake are the main risk factors for HCC. Further, excess body weight along with type 2 diabetes as components of the metabolic syndrome act as predisposing factors to the metabolic-associated fatty liver disease (non-alcoholic fatty liver disease) and progression to liver fibrosis, cirrhosis, and HCC [141,142,143,144,145,146].

Smoking is another predisposing factor for HCC [147]. The HPV impact on HCC prevalence has not been researched thoroughly. In 1992, it was suggested that infection with either HPV-16 or HPV-18 could act synergistically with HBV to promote HCC development [148]. However, to the best of the authors’ knowledge, these results have not been replicated anymore; thus, more studies are needed to provide further conclusions regarding this matter. Ma et al. conducted research in which they proved that HPV 18 E6 and E7 genes can be successfully integrated into Hep G2 cells, ultimately observing a low prevalence of HPV 16/18 in HCC samples [149]. The results of the study indicate that oncogenic HPV might constitute a cofactor acting synergistically with HBV in the onset of the HCC. HBV and HPV are DNA viruses that share a replication strategy, including the reverse transcriptase along with a characteristic life cycle that involves the integration of viral DNA into the host genome [150]. Both viruses integrate into the human telomerase reverse transcriptase (hTERT) gene in non-random sites, which might contain the genes that are altered by the viral integration event and might participate in further carcinogenesis. In addition, the viral integration site into the hTERT is involved in the determination of the tumor phenotype [151]. However, the risk of HPV as a causative agent of HCC needs further studies and verification due to a number of limited reports.

## 7. HPV and Gastric Cancer

Gastric cancer (GC) is responsible for over one million new cases along with an estimated number of 769,000 deaths in 2020 [22]. GC is a multifactorial disease. Several risk factors have been noted to have a significant impact on GC carcinogenesis including diet, smoking, family history, alcohol consumption, EBV, and *H. pylori* [152,153]. The World Cancer Research Fund/American Institute for Cancer Research (WCRF/AICR) stated that fruits and vegetables protect from GC development, whereas broiled, salt-preserved foods, and smoked foods provoke GC progression [154]. Considering alcohol intake and smoking influence on GC development, the data are consistent. Studies indicate that smokers display around an 80% increase in the risk for GC development among the population of nondrinkers. Further, individuals abusing alcohol (compared to light or moderate drinkers) are found to be at higher risk of intestinal-type non-cardia GC; a positive association was found for beer but not for wine or liquor [155,156]. It is well-established that *H. pylori* has been classified as a class I carcinogen by the World Health Organization. It directly inflames gastric mucosa and causes epigenetic effects on individual cells [157]. Except the *H. pylori* infection, *EBV* constitutes the second most relevant viral factors associated with the onset of GC. EBV-associated gastric carcinoma (EBVaGC) constitutes about 10% of all gastric carcinomas. The recruitment of the EBV-infected B-lymphocytes in the vicinity of gastric epithelia is primarily induced because the inflammation of the stomach will and might eventually increase the frequency of EBV infection of the epithelia [158,159]; in the case of the HPV relationship with gastric cancer, the data are contradictory. Similarly to EBV infection, HPV stimulates the NFκB signaling pathway crucial for the proliferation and survival of cancer cells [78]. It was also demonstrated that a promotion of EBV lytic gene expression is facilitated by the interferon regulatory factor 8 (IRF8) in complex with PU.1 [160]. Snietura et al. conducted research involving 84 surgically treated patients with gastric adenocarcinoma from Central Europe regardless of the clinical stage of the disease. All the individuals were considered negative for the highly oncogenic HPV subtypes. Having in mind the above-mentioned results, a relationship between GC and HPV infection seems doubtful considering the Central European population [161]. On the other hand, studies from China indicated that HPV infections increased the risk of GC [162]. A meta-analysis consisting of 1917 cases showed that HPV might be highly associated with a pathogenesis of GC. The pooled HPV prevalence was 28.0% (95% CI: 23.2%, 32.7%) among all the patients with GC. However, the HPV prevalence was significantly higher in Chinese patients compared to those from non-Chinese regions (31% vs. 9%, I^2^ = 95.0%, *p* < 0.001). HPV detection in the cells of GC precursor lesions (gastric dysplasia or adenoma) constitutes the only possibility to confirm any relationships [163]. Based on the currently available data, the relationship between HPV infection and GC is questionable, due to the high possibility of heterogeneity bias. More studies with an improved methodology are needed to confirm the association described above.

## 8. HPV and Colorectal Cancer

With respect to colorectal cancer (CRC), risk factors such as age over 50–60, genetic syndromes, adenomatous polyps of the colon, inflammatory bowel disease, and hereditary familial history of CRC were determined. It may also occur due to lifestyle and environmental factors including quality of nutrition, obesity, smoking, alcohol abuse, and many more [164,165]. However, a virologic component such as HPV should also be considered. HPV is associated with the development of several types of carcinomas such as cervical cancer, a subset of other anogenital cancers including vulvar, vaginal, and penile cancer, and also head and neck tumors [166,167]. Additionally, many studies have underlined the significance of HPV infection and CRC risk [168,169,170,171]. Among the more than 200 genotypes of HPVs identified, HPV 16, 18, and 33 with their highest carcinogenic capacity are the main HPV types found in colorectal cancer [172,173]. The meta-analysis of Baandrup et al. included 2630 colorectal adenocarcinomas with an HPV prevalence of 11.2%. However, the prevalence among studies ranged from 0% to 84% in the CRC and a significant geographical variation was also discovered. The highest incidence of HPV in CRC was noted in studies in South America, Asia, and the Middle East, while the lowest incidence was in North America, Europe, and Australia [174]. A significant heterogeneity among studies was also observed in Ibragimova’s meta-analysis [172]. Yet, the results of the studies investigating the presence of HPV in premalignant adenomatous polyps remain contradictory. While Cheng et al. confirmed the association between HPV and colorectal adenomas, no evidence for this coincidence was found in the Burnett-Hartman study [175,176]. The potential mechanism for HPV infection of the colorectum may consider ascending infection from anogenital sites or through hematogenous or lymphogenic spread [174]. Chen et al. investigated the expression of the E6 oncoprotein (an inactivator of p53) in HPV16 DNA-positive tumors. The study showed that the E6 oncoprotein, which was involved in CRC development, may downregulate p21 and Mdm2 transcription via inactivation of p53. In addition, it was indicated that the E6 oncoprotein was expressed in both tumor-infiltrating lymphocytes and endothelial cells of HPV16 DNA-positive colorectal tumors. This phenomenon supported the idea that the transmission of HPV to the colon might occur through peripheral blood lymphocytes [170]. It was also suggested that regarding the CRC development, a relationship between the HPV infection and new molecular biological markers of genomic instability (primarily microsatellite instability and the CpG island methylation phenotype) should be investigated [177].

Currently used HPV vaccines tuned out to be effective in preventing intraepithelial neoplasia of the cervix, vulva, vagina, and anus, which are related to HPV type 16 and 18 [19]. It was suggested that HPV vaccination not only prevents these cancers but also reduces the development of other HPV-associated cancers including CRC [170]. Therefore, there is a likelihood of a reduction in the incidence of colorectal cancer in vaccinated people [178].

## 9. HPV and Anal Cancer

Anal cancer is a rare malignancy that accounts for approximately 3% of all gastrointestinal cancers. According to current data, it is estimated that in 2020, 50,865 new cases and 19,293 deaths occurred worldwide [22]. Despite these relatively minor rates, the proper management and treatment of anal cancer are crucial as there has been an increase in the incidence over the past several decades [179,180]. Studies report that women and patients with lower socioeconomic status are more likely to present more advanced stages and more likely to die [181].

There is a close relationship between the development of anal cancer and HPV infection. Statistics show that 91% of all anal cancers are associated with this pathogen and 79% are caused only by the HPV-16 and HPV-18 subtype [182]. Subtypes HPV-39, 56, 59, 66, and 68 are considered as low-risk neoplasms. They occur in people with condylomata acuminates. However, their presence carries an increased risk of acquiring anal cancer as they are more likely to acquire high-risk HPV subtypes such as 16, 18, 31, 33, and 45 [183].

The virus penetrates the transformation zone located in the rectal columnar mucosa, distal to the dentate line, and escalates proximally from the squamocolumnar junction [184]. The initiation of the oncogenesis process is due to E6 and E7 proteins produced by the virus. These components inactivate the function of two tumor suppressors, p53 and retinoblastoma protein (pRb). As a result, repair mechanisms are stopped and the dysregulated cell cycle promotes tumor progression [37,170].

There are several risk factors for anal cancer such as older age, smoking, or immunosuppression. However, the greatest risk is seen in individuals who have multiple sexual partners and practice promiscuous sexual behaviors [185].

## 10. HPV Vaccination

Globally, 5% of all human cancers are attributable to HPV, including cervical, other anogenital, as well as head and neck cancers. Together, they pose a serious problem of 630,000 new cancer cases per year worldwide [186]. HPVs are DNA tumor viruses with more than 200 genotypes described. They are categorized into two groups—‘high-risk’ or ‘low-risk’ HPV types [187]. HPV-16 and -18, which belong to the first group, are the most prevalent types associated with cervical cancer but also with abovementioned colorectal and anal cancers [98,172,182].

As it comes to pathogenesis, HPV infects the basal epithelial cells, which may occur in microlesions of the skin or mucosa. The entry of the virus and its genome into the nucleus of the infected cell is possible due to the attachment of L1 and L2 capsid proteins to epithelial cell receptors [2]. The integration of HPV DNA into the host cell genome is a key event in carcinogenesis. Proteins that are first expressed may regulate the host cell life cycle and genome replication, leading to oncogenes amplification and the disruption of tumor suppressor genes [188]. E6 and E7, which are specific viral oncoproteins, play the main role by interfering with two essential tumor suppressor genes: host apoptosis regulator protein p53 and pRb. As a result, the cell cycle regulation is disrupted and the host cell life is prolonged, leading to genomic instability and ultimately to cancer [189].

Fortunately, HPV prophylactic vaccines, which are based on recombinantly expressed virus-like particles (VLPs), were found to be effective at preventing infection and neoplastic disease [188]. According to Castle et al., the inactive HPV L1 VLPs produce neutralizing antibodies against targeted HPV types and elicit a strong humoral immune response at the same time. As an effect, the entrance of viral particles into host cells is blocked by the binding antibodies. Additionally, the immune response invoked by HPV VLPs was stronger than the one induced by natural infection [190]. Although these vaccines block initial infection by certain HPV types, they are not effective at eliminating pre-existing infections. This is because L1 capsid proteins, which are the target antigens, are not expressed in the infected basal epithelial cells [191]. Interestingly, immunization at younger age resulted in higher antibody titers than at older age [190].

HPV vaccines development started at the beginning of the early 1990s. Currently, there are three available HPV vaccines including bivalent Cervarix, quadrivalent Gardasil, and nonavalent Gardasil9. Cervarix protects against two high-risk types, HPV 16 and 18, while Gardasil is also directed against two low-risk types, HPV 6 and 11. Gardasil9 targets the four HPV types (6, 11, 16, 18) that are in the quadrivalent HPV vaccine and five additional oncogenic types including type 31, 33, 45, 52, and 58 [192]. Additionally, HPV vaccination is recommended due to its proven effectiveness, cost-effectiveness, and safety profile [193].

## 11. Conclusions

Persistent HPV infection targets immune signaling as well as tumor suppression pathways, eventually leading to the induction of oncogenic promotion in the form of tumors primarily located within the cervix, vulva, head and neck, and anus. Although HPV infection is mostly recognized as being associated with gynecologic tumors, the increasing amount of research also suggests its association with GI cancers; amongst these, most of the data concern anal cancer, indicating a clear relationship between HPV infection and the possible induction and progression of anal cancerous lesions. HPV infection is most likely to facilitate the worsening of the course of other concomitant infections (either bacterial or viral) that, together with other coexisting infections by highly oncogenic bacteria and viruses, might include precancerous alterations, ultimately leading to cancer. The aspect of concomitant viral and bacterial diseases that are potentially oncogenic is crucial regarding the discussion about the potential oncogenic effects of HPV infection and its association with GI cancers. Based on the current state of knowledge, the relationship between HPV infection and the onset of esophageal, liver, colorectal, and gastric cancer cannot be clearly established, due to many contradictory research data. What should further be evaluated as well is the association between HPV vaccination and the risk of GI cancers as, except for gynecological cancers, it was suggested that the HPV vaccine might also minimize the risk of CRC. Nevertheless, much research suggests strong evidence for a potential association between HPV infection and either the induction or progression of GI cancers, which should be further evaluated via more research with improved methodological tools and on the greatest number of patients as possible.

## Figures and Tables

**Table 1:** Oncogenic proteins of HPV and their mechanisms of action.

Protein	Mechanism of Action	Effect	References
E6	Binding to cellular ubiquitin ligase E6-associated protein (E6-AP) and E6/E6AP/p53 complex formation	Promotion of p53 degradation	[64,84]
	Binding to the cellular proteins containing PSD-95/DLG/ZO-1 (PDZ) domains	Proteolytic degradation of potential tumor suppressor proteins, such as Dlg, Scribble, and MAGI-1	[85]
	Binding to myc proteins and inducing expression of the human telomerase reverse transcriptase (hTERT) catalytic subunit	Telomerase activation leading to prolonged life of HPV-16 infected cells	[73,75]
	Binding to NFX-1 isoforms: NFX1-123 and NFX1-91	Increase in hTERT mRNA levels and degradation of NFX1-91 telomerase repressor	[75,77]
	Binding to the Fas-associated Death Domain (FADD)	Prevention of Fas-induced apoptosis	[86]
	Stimulating pro-apoptotic Bak degradation by E6AP protein	Inhibition of Bak-induced apoptosis	[87,88]
	Bax protein inhibition and reduction in Bax mRNA expression	Inhibition of Bax-induced apoptosis	[89,90]
	Dysregulation of cellular microRNAs (miRNAs)	Dysregulation of cycle regulation, apoptosis, cell–cell adhesion, cell mobility, and proliferation	[91,92,93]
	Increasing the level of miR-20b	Inducing morphological cell alterations	[94]
E7	Proteasomal degradation of the pRB/E2F repressor complex	Activation of genes necessary for S-phase progression	[48]
	Binding to p107 and p130		
	Abrogation of the inhibitory activities of the CKIs p21^CIP1^ and p27^KIP1^	Dysregulation the G1/S-phase transition	[95,96,97,98]
	Overexpression of cyclins E and A—the regulatory subunits of cdk2		
	Inhibition of TGFβ signaling	Impaired cellular differentiation	[99,100]
	Binding to PTPN14		
	Binding with class I histone deacetylases (HDACs)	Invalid chromatin remodeling	[101]
	Binding to KDM6A/B	Cellular histone modifications	[102,103]
	Binding to DNMT1	Epigenetic dysregulation	[104]
	Binding to IRF1, IRF9	Repression of the innate antiviral immune response	[105,106,107,108,109]
	Secretion of IL-18BP		
	Inhibition of the TLR9 and cGAS-STING signaling axis		
	Activation of the ATM and ATR pathways	Genome instability, aberrant centrosome duplication	[61,110,111]
	γ-tubulin disturbance		

## Abbreviations

ASIR	age-standardized incidence rate
CCRT	concurrent chemoradiotherapy
CRC	colorectal cancer
EAC	esophageal adenocarcinoma
EBV	*Epstein–Barr Virus*
ESCC	esophageal squamous cell carcinoma
GC	gastric cancer
GIT	gastrointestinal tract
HBV	hepatitis B virus
HCC	hepatocellular carcinoma
HCV	hepatitis C virus
HPV	*Human Papilloma Virus*
*H. pylori*	*Helicobacter pylori*
hTERT	human telomerase reverse transcriptase
IARC	International Agency for Research on Cancer
LCR	long control region
STI	sexually transmitted infection

## References

[1] Bzhalava D., Guan P., Franceschi S., Dillner J., Clifford G. (2013). A systematic review of the prevalence of mucosal and cutaneous human papillomavirus types. Virology.

[2] zur Hausen H. (2002). Papillomaviruses and cancer: From basic studies to clinical application. Nat. Rev. Cancer.

[3] Muñoz N., Bosch F.X., De Sanjosé S., Herrero R., Castellsagué X., Shah K.V., Snijders P.J.F., Meijer C.J.L.M. (2003). Epidemiologic Classification of Human Papillomavirus Types Associated with Cervical Cancer. N. Engl. J. Med..

[4] Egawa N., Doorbar J. (2017). The low-risk papillomaviruses. Virus Res..

[5] Butts B.N., Fischer P.R., Mack K.J. (2017). Human Papillomavirus Vaccine and Postural Orthostatic Tachycardia Syndrome: A Review of Current Literature. J. Child Neurol..

[6] Tommasino M. (2014). The human papillomavirus family and its role in carcinogenesis. Semin. Cancer Biol..

[7] Brouwer A.F., Eisenberg M.C., Meza R. (2018). Case Studies of Gastric, Lung, and Oral Cancer Connect Etiologic Agent Prevalence to Cancer Incidence. Cancer Res..

[8] Ferlay J., Shin H.-R., Bray F., Forman D., Mathers C., Parkin D.M. (2010). Estimates of worldwide burden of cancer in 2008: GLOBOCAN 2008. Int. J. Cancer.

[9] Forman D., Burley V. (2006). Gastric cancer: Global pattern of the disease and an overview of environmental risk factors. Best Pr. Res. Clin. Gastroenterol..

[10] Baj J., Korona-Głowniak I., Forma A., Maani A., Sitarz E., Rahnama-Hezavah M., Radzikowska E., Portincasa P. (2020). Mechanisms of the Epithelial–Mesenchymal Transition and Tumor Microenvironment in Helicobacter pylori-Induced Gastric Cancer. Cells.

[11] Machlowska J., Baj J., Sitarz M., Maciejewski R., Sitarz R. (2020). Gastric Cancer: Epidemiology, Risk Factors, Classification, Genomic Characteristics and Treatment Strategies. Int. J. Mol. Sci..

[12] Baj J., Forma A., Sitarz M., Portincasa P., Garruti G., Krasowska D., Maciejewski R. (2020). *Helicobacter pylori* Virulence Factors—Mechanisms of Bacterial Pathogenicity in the Gastric Microenvironment. Cells.

[13] Jemal A., Bray F., Ferlay J. (2008). Global Cancer Statistics: 2011. CA Cancer J. Clin..

[14] Zheng Z.-M. (2006). Papillomavirus genome structure, expression, and post-transcriptional regulation. Front. Biosci..

[15] Bzhalava D., Eklund C., Dillner J. (2015). International standardization and classification of human papillomavirus types. Virology.

[16] de Villiers E.-M. (2013). Cross-roads in the classification of papillomaviruses. Virology.

[17] Haley C.T., Mui U.N., Vangipuram R., Rady P.L., Tyring S.K. (2019). Human oncoviruses: Mucocutaneous manifestations, pathogenesis, therapeutics, and prevention. J. Am. Acad. Dermatol..

[18] Doorbar J., Quint W., Banks L., Bravo I.G., Stoler M., Broker T.R., Stanley M.A. (2012). The Biology and Life-Cycle of Human Papillomaviruses. Vaccine.

[19] Barrow-Laing L., Chen W., Roman A. (2010). Low- and high-risk human papillomavirus E7 proteins regulate p130 differently. Virology.

[20] Bouvard V., Baan R., Straif K., Grosse Y., Secretan B., El Ghissassi F., Benbrahim-Tallaa L., Guha N., Freeman C., Galichet L. (2009). A review of human carcinogens—Part B: Biological agents. Lancet Oncol..

[21] de Martel C., Georges D., Bray F., Ferlay J., Clifford G.M. (2020). Global burden of cancer attributable to infections in 2018: A worldwide incidence analysis. Lancet Glob. Health.

[22] Sung H., Ferlay J., Siegel R.L., Laversanne M., Soerjomataram I., Jemal A., Bray F. (2021). Global Cancer Statistics 2020: GLOBOCAN Estimates of Incidence and Mortality Worldwide for 36 Cancers in 185 Countries. CA Cancer J. Clin..

[23] Smith J.S., Lindsay L., Hoots B., Keys J., Franceschi S., Winer R., Clifford G.M. (2007). Human papillomavirus type distribution in invasive cervical cancer and high-grade cervical lesions: A meta-analysis update. Int. J. Cancer.

[24] Kreimer A.R., Clifford G.M., Boyle P., Franceschi S. (2005). Human Papillomavirus Types in Head and Neck Squamous Cell Carcinomas Worldwide: A Systematic Review. Cancer Epidemiol. Biomark. Prev..

[25] de Sanjosé S., Brotons M., Pavon M.A. (2018). The natural history of human papillomavirus infection. Best Pr. Res. Clin. Obstet. Gynaecol..

[26] Lehoux M., D’Abramo C.M., Archambault J. (2009). Molecular mechanisms of human papillomavirus-induced carcinogenesis. Public Health Genom..

[27] Balaji R., MacCosham A., Williams K., El-Zein M., Franco E.L. (2020). Directionality of Genital Human Papillomavirus Infection Transmission Within Heterosexual Couples: A Systematic Review and Meta-analysis. J. Infect. Dis..

[28] Malagón T., MacCosham A., Burchell A.N., El-Zein M., Tellier P.-P., Coutlée F., Franco E.L., for the HITCH Study Group (2021). Sex- and Type-specific Genital Human Papillomavirus Transmission Rates Between Heterosexual Partners: A Bayesian Reanalysis of the HITCH Cohort. Epidemiology.

[29] Kombe A.J.K., Li B., Zahid A., Mengist H.M., Bounda G.-A., Zhou Y., Jin T. (2021). Epidemiology and Burden of Human Papillomavirus and Related Diseases, Molecular Pathogenesis, and Vaccine Evaluation. Front. Public Health.

[30] Sabeena S.P., Bhat P., Kamath V., Arunkumar G. (2017). Possible non-sexual modes of transmission of human papilloma virus. J. Obstet. Gynaecol. Res..

[31] Chatzistamatiou K., Sotiriadis A., Agorastos T. (2015). Effect of mode of delivery on vertical human papillomavirus transmission—A meta-analysis. J. Obstet. Gynaecol..

[32] Liu Z., Rashid T., Nyitray A.G. (2016). Penises not required: A systematic review of the potential for human papillomavirus horizontal transmission that is non-sexual or does not include penile penetration. Sex. Health.

[33] Arnold M., Abnet C.C., Neale R.E., Vignat J., Giovannucci E.L., McGlynn K.A., Bray F. (2020). Global Burden of 5 Major Types of Gastrointestinal Cancer. Gastroenterology.

[34] Giroglou T., Florin L., Schäfer F., Streeck R.E., Sapp M. (2001). Human Papillomavirus Infection Requires Cell Surface Heparan Sulfate. J. Virol..

[35] Pinidis P., Tsikouras P., Iatrakis G., Zervoudis S., Koukouli Z., Bothou A., Galazios G., Vladareanu S. (2016). Human Papilloma Virus’ Life Cycle and Carcinogenesis. Maedica-J. Clin. Med..

[36] Egawa N., Egawa K., Griffin H.M., Doorbar J. (2015). Human Papillomaviruses; Epithelial Tropisms, and the Development of Neoplasia. Viruses.

[37] Schiffman M., Doorbar J., Wentzensen N., De Sanjosé S., Fakhry C., Monk B.J., Stanley M.A., Franceschi S. (2016). Carcinogenic human papillomavirus infection. Nat. Rev. Dis. Prim..

[38] Albert E., Laimins L. (2020). Regulation of the Human Papillomavirus Life Cycle by DNA Damage Repair Pathways and Epigenetic Factors. Viruses.

[39] Kanda T., Furuno A., Yoshiike K. (1988). Human papillomavirus type 16 open reading frame E7 encodes a transforming gene for rat 3Y1 cells. J. Virol..

[40] Watanabe S., Yoshiike K. (1988). Transformation of rat 3Y1 cells by human papillomavirus type-18 DNA. Int. J. Cancer.

[41] Vousden K.H., Doniger J., Dipaolo J.A., Lowy D.R. (1988). The E7 open reading frame of human papillomavirus type 16 encodes a transforming gene. Oncogene Res..

[42] Tanaka A., Noda T., Yajima H., Hatanaka M., Ito Y. (1989). Identification of a transforming gene of human papillomavirus type 16. J. Virol..

[43] Phelps W.C., Yee C.L., Munger K., Howley P. (1988). The human papillomavirus type 16 E7 gene encodes transactivation and transformation functions similar to those of adenovirus E1A. Cell.

[44] Yasumoto S., Burkhardt A.L., Doniger J., DiPaolo J.A. (1986). Human papillomavirus type 16 DNA-induced malignant transformation of NIH 3T3 cells. J. Virol..

[45] Hudson J.B., Bedell M.A., McCance D.J., Laiminis L.A. (1990). Immortalization and altered differentiation of human keratinocytes in vitro by the E6 and E7 open reading frames of human papillomavirus type 18. J. Virol..

[46] Hawley-Nelson P., Vousden K., Hubbert N., Lowy D., Schiller J. (1989). HPV16 E6 and E7 proteins cooperate to immortalize human foreskin keratinocytes. EMBO J..

[47] Münger K., Phelps W.C., Bubb V., Howley P.M., Schlegel R. (1989). The E6 and E7 genes of the human papillomavirus type 16 together are necessary and sufficient for transformation of primary human keratinocytes. J. Virol..

[48] McLaughlin-Drubin M.E., Münger K. (2009). The human papillomavirus E7 oncoprotein. Virology.

[49] Dyson N., Howley P.M., Münger K., Harlow E. (1989). The Human Papilloma Virus-16 E7 Oncoprotein Is Able to Bind to the Retinoblastoma Gene Product. Science.

[50] Davies R., Hicks R., Crook T., Morris J., Vousden K. (1993). Human papillomavirus type 16 E7 associates with a histone H1 kinase and with p107 through sequences necessary for transformation. J. Virol..

[51] Dyson N., Guida P., Münger K., Harlow E. (1992). Homologous sequences in adenovirus E1A and human papillomavirus E7 proteins mediate interaction with the same set of cellular proteins. J. Virol..

[52] Hiebert S.W., Chellappan S.P., Horowitz J.M., Nevins J.R. (1992). The interaction of RB with E2F coincides with an inhibition of the transcriptional activity of E2F. Genes Dev..

[53] Ganguly N., Parihar S.P. (2009). Human papillomavirus E6 and E7 oncoproteins as risk factors for tumorigenesis. J. Biosci..

[54] Frolov M.V., Dyson N.J. (2004). Molecular mechanisms of E2F-dependent activation and pRB-mediated repression. J. Cell Sci..

[55] Nevins J.R. (2001). The Rb/E2F pathway and cancer. Hum. Mol. Genet..

[56] Boyer S.N., Wazer D.E., Band V. (1996). E7 protein of human papilloma virus-16 induces degradation of retinoblastoma protein through the ubiquitin-proteasome pathway. Cancer Res..

[57] Huh K., Zhou X., Hayakawa H., Cho J.-Y., Libermann T.A., Jin J., Harper J.W., Munger K. (2007). Human Papillomavirus Type 16 E7 Oncoprotein Associates with the Cullin 2 Ubiquitin Ligase Complex, Which Contributes to Degradation of the Retinoblastoma Tumor Suppressor. J. Virol..

[58] Duensing S., Lee L.Y., Duensing A., Basile J., Piboonniyom S.-O., Gonzalez S., Crum C.P., Münger K. (2000). The human papillomavirus type 16 E6 and E7 oncoproteins cooperate to induce mitotic defects and genomic instability by uncoupling centrosome duplication from the cell division cycle. Proc. Natl. Acad. Sci. USA.

[59] Duensing S., Münger K. (2003). Human Papillomavirus Type 16 E7 Oncoprotein Can Induce Abnormal Centrosome Duplication through a Mechanism Independent of Inactivation of Retinoblastoma Protein Family Members. J. Virol..

[60] Korzeniewski N., Treat B., Duensing S. (2011). The HPV-16 E7 oncoprotein induces centriole multiplication through deregulation of Polo-like kinase 4 expression. Mol. Cancer.

[61] Nguyen C.L., Eichwald C., Nibert M.L., Münger K. (2007). Human Papillomavirus Type 16 E7 Oncoprotein Associates with the Centrosomal Component γ-Tubulin. J. Virol..

[62] Nguyen C.L., Munger K. (2009). Human Papillomavirus E7 Protein Deregulates Mitosis via an Association with Nuclear Mitotic Apparatus Protein 1. J. Virol..

[63] Werness B.A., Levine A.J., Howley P.M. (1990). Association of Human Papillomavirus Types 16 and 18 E6 Proteins with p53. Science.

[64] Scheffner M., Werness B.A., Huibregtse J.M., Levine A.J., Howley P.M. (1990). The E6 oncoprotein encoded by human papillomavirus types 16 and 18 promotes the degradation of p53. Cell.

[65] Huibregtse J.M., Scheffner M., Howley P.M. (1991). A cellular protein mediates association of p53 with the E6 oncoprotein of human papillomavirus types 16 or 18. EMBO J..

[66] Huibregtse J.M., Scheffner M., Howley P.M. (1993). Localization of the E6-AP regions that direct human papillomavirus E6 binding, association with p53, and ubiquitination of associated proteins. Mol. Cell. Biol..

[67] Kuballa P., Matentzoglu K., Scheffner M. (2007). The Role of the Ubiquitin Ligase E6-AP in Human Papillomavirus E6-mediated Degradation of PDZ Domain-containing Proteins. J. Biol. Chem..

[68] Hengstermann A., D’Silva M.A., Kuballa P., Butz K., Hoppe-Seyler F., Scheffner M. (2005). Growth Suppression Induced by Downregulation of E6-AP Expression in Human Papillomavirus-Positive Cancer Cell Lines Depends on p53. J. Virol..

[69] Tomaić V., Pim D., Banks L. (2009). The stability of the human papillomavirus E6 oncoprotein is E6AP dependent. Virology.

[70] Shai A., Pitot H.C., Lambert P.F. (2010). E6-Associated Protein Is Required for Human Papillomavirus Type 16 E6 to Cause Cervical Cancer in Mice. Cancer Res..

[71] Massimi P., Shai A., Lambert P., Banks L. (2007). HPV E6 degradation of p53 and PDZ containing substrates in an E6AP null background. Oncogene.

[72] Liu Y., Chen J.J., Gao Q., Dalal S., Hong Y., Mansur C.P., Band V., Androphy E.J. (1999). Multiple Functions of Human Papillomavirus Type 16 E6 Contribute to the Immortalization of Mammary Epithelial Cells. J. Virol..

[73] Klingelhutz A., Foster S.A., McDougall J.K. (1996). Telomerase activation by the E6 gene product of human papillomavirus type 16. Nature.

[74] Cong Y.-S., Wright W.E., Shay J.W. (2002). Human Telomerase and Its Regulation. Microbiol. Mol. Biol. Rev..

[75] Wu K.-J., Grandori C., Amacker M., Simon-Vermot N., Polack A., Lingner J., Dalla-Favera R. (1999). Direct activation of TERT transcription by c-MYC. Nat. Genet..

[76] Gewin L., Myers H., Kiyono T., Galloway D.A. (2004). Identification of a novel telomerase repressor that interacts with the human papillomavirus type-16 E6/E6-AP complex. Genes Dev..

[77] Katzenellenbogen R.A., Vliet-Gregg P., Xu M., Galloway D.A. (2009). NFX1-123 Increases hTERT Expression and Telomerase Activity Posttranscriptionally in Human Papillomavirus Type 16 E6 Keratinocytes. J. Virol..

[78] Morgan E.L., Macdonald A. (2019). Autocrine STAT3 activation in HPV positive cervical cancer through a virus-driven Rac1—NFκB—IL-6 signalling axis. PLoS Pathog..

[79] Hong S., Laimins L.A. (2013). The JAK-STAT Transcriptional Regulator, STAT-5, Activates the ATM DNA Damage Pathway to Induce HPV 31 Genome Amplification upon Epithelial Differentiation. PLoS Pathog..

[80] Morgan E.L., Scarth J.A., Patterson M.R., Wasson C.W., Hemingway G.C., Barba-Moreno D., Macdonald A. (2020). E6-mediated activation of JNK drives EGFR signalling to promote proliferation and viral oncoprotein expression in cervical cancer. Cell Death Differ..

[81] He C., Mao D., Hua G., Lv X., Chen X., Angeletti P.C., Dong J., Remmenga S.W., Rodabaugh K.J., Zhou J. (2015). The Hippo/YAP pathway interacts with EGFR signaling and HPV oncoproteins to regulate cervical cancer progression. EMBO Mol. Med..

[82] Morgan E.L., Patterson M.R., Ryder E.L., Lee S.Y., Wasson C., Harper K.L., Li Y., Griffin S., Blair G.E., Whitehouse A. (2020). MicroRNA-18a targeting of the STK4/MST1 tumour suppressor is necessary for transformation in HPV positive cervical cancer. PLoS Pathog..

[83] Scarth J.A., Patterson M.R., Morgan E.L., Macdonald A. (2021). The human papillomavirus oncoproteins: A review of the host pathways targeted on the road to transformation. J. Gen. Virol..

[84] Martinez-Zapien D., Ruiz F.X., Poirson J., Mitschler A., Ramirez J., Forster A., Cousido-Siah A., Masson M., Vande Pol S., Podjarny A. (2016). Structure of the E6/E6AP/p53 complex required for HPV-mediated degradation of p53. Nature.

[85] Hoppe-Seyler K., Bossler F., Braun J.A., Herrmann A.L., Hoppe-Seyler F. (2018). The HPV E6/E7 Oncogenes: Key Factors for Viral Carcinogenesis and Therapeutic Targets. Trends Microbiol..

[86] Filippova M., Parkhurst L., Duerksen-Hughes P. (2004). The Human Papillomavirus 16 E6 Protein Binds to Fas-associated Death Domain and Protects Cells from Fas-triggered Apoptosis. J. Biol. Chem..

[87] Thomas M., Banks L. (1998). Inhibition of Bak-induced apoptosis by HPV-18 E6. Oncogene.

[88] Thomas M., Banks L. (1999). Human papillomavirus (HPV) E6 interactions with Bak are conserved amongst E6 proteins from high and low risk HPV types. J. Gen. Virol..

[89] Magal S.S., Jackman A., Ish-Shalom S., Botzer L.E., Gonen P., Schlegel R., Sherman L. (2005). Downregulation of Bax mRNA expression and protein stability by the E6 protein of human papillomavirus 16. J. Gen. Virol..

[90] Vogt M., Butz K., Dymalla S., Semzow J., Hoppe-Seyler F. (2006). Inhibition of Bax activity is crucial for the antiapoptotic function of the human papillomavirus E6 oncoprotein. Oncogene.

[91] Suzuki H., Maruyama R., Yamamoto E., Kai M. (2012). DNA methylation and microRNA dysregulation in cancer. Mol. Oncol..

[92] Harden M.E., Prasad N., Griffiths A., Munger K. (2017). Modulation of microRNA-mRNA Target Pairs by Human Papillomavirus 16 Oncoproteins. mBio.

[93] Yablonska S., Hoskins E.E., Wells S.I., Khan S.A. (2013). Identification of miRNAs Dysregulated in Human Foreskin Keratinocytes (HFKs) Expressing the Human Papillomavirus (HPV) Type 16 E6 and E7 Oncoproteins. MicroRNA.

[94] Cheng Y., Geng L., Zhao L., Zuo P., Wang J. (2017). Human papillomavirus E6-regulated microRNA-20b promotes invasion in cervical cancer by targeting tissue inhibitor of metalloproteinase 2. Mol. Med. Rep..

[95] Zerfass K., Schulze A., Spitkovsky D., Friedman V., Henglein B., Jansen-Dürr P. (1995). Sequential activation of cyclin E and cyclin A gene expression by human papillomavirus type 16 E7 through sequences necessary for transformation. J. Virol..

[96] Funk J.O., Waga S., Harry J.B., Espling E., Stillman B., Galloway D.A. (1997). Inhibition of CDK activity and PCNA-dependent DNA replication by p21 is blocked by interaction with the HPV-16 E7 oncoprotein. Genes Dev..

[97] Zerfass-Thome K., Zwerschke W., Mannhardt B., Tindle R., Botz J.W., Jansen-Dürr P. (1996). Inactivation of the cdk inhibitor p27KIP1 by the human papillomavirus type 16 E7 oncoprotein. Oncogene.

[98] Moody C.A., Laimins L.A. (2010). Human papillomavirus oncoproteins: Pathways to transformation. Nat. Cancer.

[99] White E.A., Munger K., Howley P. (2016). High-Risk Human Papillomavirus E7 Proteins Target PTPN14 for Degradation. mBio.

[100] Pietenpol J.A., Stein R.W., Moran E., Yaciuk P., Schlegel R., Lyons R.M., Pittelkow M.R., Munger K., Howley P., Moses H.L. (1990). TGF-β1 inhibition of c-myc transcription and growth in keratinocytes is abrogated by viral transforming proteins with pRB binding domains. Cell.

[101] Brehm A., Nielsen S.J., Miska E.A., McCance D.J., Reid J.L., Bannister A.J., Kouzarides T. (1999). The E7 oncoprotein associates with Mi2 and histone deacetylase activity to promote cell growth. EMBO J..

[102] Cigno I.L., Calati F., Borgogna C., Zevini A., Albertini S., Martuscelli L., De Andrea M., Hiscott J., Landolfo S., Gariglio M. (2020). Human Papillomavirus E7 Oncoprotein Subverts Host Innate Immunity via SUV39H1-Mediated Epigenetic Silencing of Immune Sensor Genes. J. Virol..

[103] Wu L., Cao J., Cai W.L., Lang S.M., Horton J.R., Jansen D.J., Liu Z.Z., Chen J.F., Zhang M., Mott B.T. (2018). KDM5 histone demethylases repress immune response via suppression of STING. PLoS Biol..

[104] Burgers W.A., Blanchon L., Pradhan S., de Launoit Y., Kouzarides T., Fuks F. (2006). Viral oncoproteins target the DNA methyltransferases. Oncogene.

[105] Barnard P., McMillan N. (1999). The Human Papillomavirus E7 Oncoprotein Abrogates Signaling Mediated by Interferon-α. Virology.

[106] Park J.-S., Kim E.-J., Kwon H.-J., Hwang E.-S., Namkoong S.-E., Um S.-J. (2000). Inactivation of Interferon Regulatory Factor-1 Tumor Suppressor Protein by HPV E7 Oncoprotein. J. Biol. Chem..

[107] Hasan U.A., Zannetti C., Parroche P., Goutagny N., Malfroy M., Roblot G., Carreira C., Hussain I., Müller M., Taylor-Papadimitriou J. (2013). The Human papillomavirus type 16 E7 oncoprotein induces a transcriptional repressor complex on the Toll-like receptor 9 promoter. J. Exp. Med..

[108] Richards K.H., Doble R., Wasson C.W., Haider M., Blair G.E., Wittmann M., Macdonald A. (2014). Human Papillomavirus E7 Oncoprotein Increases Production of the Anti-Inflammatory Interleukin-18 Binding Protein in Keratinocytes. J. Virol..

[109] Lau L., Gray E.E., Brunette R.L., Stetson D.B. (2015). DNA tumor virus oncogenes antagonize the cGAS-STING DNA-sensing pathway. Science.

[110] Hong S., Cheng S., Iovane A., Laimins L.A. (2015). STAT-5 Regulates Transcription of the Topoisomerase IIβ-Binding Protein 1 (TopBP1) Gene To Activate the ATR Pathway and Promote Human Papillomavirus Replication. mBio.

[111] Blackford A.N., Jackson S.P. (2017). ATM, ATR, and DNA-PK: The Trinity at the Heart of the DNA Damage Response. Mol. Cell.

[112] Nees M., Geoghegan J., Munson P., Munson P., Prabhu V., Liu Y., Androphy E., Woodworth C.D. (2000). Human Papillomavirus Type 16 E6 and E7 Proteins Inhibit Differentia-tion-dependent Expression of Transforming Growth Factor-β2 in Cervical Keratinocytes. Cancer Res..

[113] Melar-New M., Laimins L.A. (2010). Human Papillomaviruses Modulate Expression of MicroRNA 203 upon Epithelial Differentiation to Control Levels of p63 Proteins. J. Virol..

[114] Dillner J. (1999). The serological response to papillomaviruses. Semin. Cancer Biol..

[115] Molijn A., Kleter B., Quint W., van Doorn L.-J. (2005). Molecular diagnosis of human papillomavirus (HPV) infections. J. Clin. Virol..

[116] FDA-Approved HPV Tests—LabCE.com, Laboratory Continuing Education. https://www.labce.com/spg761630_fda_approved_hpv_tests.aspx.

[117] Lie A.K., Kristensen G. (2008). Human papillomavirus E6/E7 mRNA testing as a predictive marker for cervical carcinoma. Expert Rev. Mol. Diagn..

[118] Evans M.F., Cooper K. (2003). Human papillomavirus integration: Detection byin situ hybridization and potential clinical application. J. Pathol..

[119] Leto M.D.G.P., Júnior G.F.D.S., Porro A.M., Tomimori J. (2011). Infecção pelo papilomavírus humano: Etiopatogenia, biologia molecular e manifestações clínicas. An. Bras. De Dermatol..

[120] Nuovo G.J. (2003). Diagnosis of Human Papillomavirus Using In Situ Hybridization and In Situ Polymerase Chain Reaction. Methods Mol. Biol..

[121] Tsakogiannis D., Gartzonika C., Levidiotou-Stefanou S., Markoulatos P. (2017). Molecular approaches for HPV genotyping and HPV-DNA physical status. Expert Rev. Mol. Med..

[122] Qureishi A., Mawby T., Fraser L., Shah K.A., Møller H., Winter S. (2017). Current and future techniques for human papilloma virus (HPV) testing in oropharyngeal squamous cell carcinoma. Eur. Arch. Oto-Rhino-Laryngol..

[123] Paver E.C., Currie A.M., Gupta R., Dahlstrom J.E. (2019). Human papilloma virus related squamous cell carcinomas of the head and neck: Diagnosis, clinical implications and detection of HPV. Pathology.

[124] Döberitz M.V.K. (2016). The causal role of human papillomavirus infections in non-anogenital cancers. It’s time to ask for the *functional evidence*. Int. J. Cancer.

[125] Prigge E.S., Doeberitz M.V.K., Reuschenbach M. (2016). Clinical relevance and implications of HPV-induced neoplasia in different anatomical locations. Mutat. Res. Mutat. Res..

[126] Torre L.A., Siegel R.L., Ward E.M., Jemal A. (2016). Global Cancer Incidence and Mortality Rates and Trends—An Update. Cancer Epidemiol. Biomark. Prev..

[127] Znaor A., Brennan P., Gajalakshmi V., Mathew A., Shanta V., Varghese C., Boffetta P. (2003). Independent and combined effects of tobacco smoking, chewing and alcohol drinking on the risk of oral, pharyngeal and esophageal cancers in Indian men. Int. J. Cancer.

[128] Ryan A.M., Duong M., Healy L., Ryan S.A., Parekh N., Reynolds J.V., Power D.G. (2011). Obesity, metabolic syndrome and esophageal ade-nocarcinoma: Epidemiology, etiology and new targets. Cancer Epidemiol..

[129] Zhang S.-K., Guo L.-W., Chen Q., Zhang M., Liu S.-Z., Quan P.-L., Lu J.-B., Sun X.-B. (2015). The association between human papillomavirus 16 and esophageal cancer in Chinese population: A meta-analysis. BMC Cancer.

[130] Wang J., Zhao L., Yan H., Che J., Huihui L., Jun W., Liu B., Cao B. (2016). A Meta-Analysis and Systematic Review on the Association between Human Papillomavirus (Types 16 and 18) Infection and Esophageal Cancer Worldwide. PLoS ONE.

[131] Koshiol J., Wei W.Q., Kreimer A.R., Chen W., Gravitt P., Ren J.S., Abnet C.C., Wang J.B., Kamangar F., Lin D.M. (2010). No role for human papillomavirus in esophageal squa-mous cell carcinoma in China. Int. J. Cancer.

[132] Halec G., Schmitt M., Egger S., Abnet C.C., Babb C., Dawsey S.M., Flechtenmacher C., Gheit T., Hale M., Holzinger D. (2016). Mucosal Alpha-Papillomaviruses are not associated with EsophagealSquamous Cell Carcinomas: Lack of Mechanistic Evidence from South Africa, Chinaand Iran and from a World-Wide Meta-Analysis. Int. J. Cancer.

[133] Rajendra S., Wang B., Snow E.T., Sharma P., Pavey D., Merrett N., Ball M.J., Brain T., Fernando R., Robertson I.K. (2013). Transcriptionally Active Human Papillomavirus Is Strongly Associated With Barrett’s Dysplasia and Esophageal Adenocarcinoma. Am. J. Gastroenterol..

[134] Rajendra S., Wang B., Merrett N., Sharma P., Humphris J., Lee H.C., Wu J. (2015). Genomic analysis of HPV-positive versus HPV-negative oesophageal adenocarcinoma identifies a differential mutational landscape. J. Med Genet..

[135] Agalliu I., Chen Z., Wang T., Hayes R.B., Freedman N.D., Gapstur S.M., Burk R.D. (2018). Oral Alpha, Beta, and Gamma HPV Types and Risk of Incident Esophageal Cancer. Cancer Epidemiol. Biomark. Prev..

[136] Guo L., Liu S., Zhang S., Chen Q., Zhang M., Quan P., Sun X.B. (2016). uman papillomavirus-related esophageal cancer survival: A systematic review and meta-analysis. Medicine.

[137] Huang F.-L., Yu S.-J. (2018). Esophageal cancer: Risk factors, genetic association, and treatment. Asian J. Surg..

[138] van Hagen P., Hulshof M.C.C.M., Van Lanschot J.J.B., Steyerberg E.W., van Berge Henegouwen M.I., Wijnhoven B.P.L., Richel D.J., Nieuwenhuijzen G.A.P., Hospers G.A.P., Bonenkamp J.J. (2012). Preoperative Chemoradiotherapy for Esophageal or Junctional Cancer. N. Engl. J. Med..

[139] Bognár L., Hegedűs I., Bellyei S., Pozsgai É., Zoltán L., Gombos K., Horváth Ö.P., Vereczkei A., Papp A. (2018). Prognostic role of HPV infection in esophageal squamous cell carcinoma. Infect. Agent. Cancer.

[140] Baj J., Bryliński Ł., Woliński F., Granat M., Kostelecka K., Duda P., Flieger J., Teresiński G., Buszewicz G., Furtak-Niczyporuk M. (2022). Biomarkers and Genetic Markers of Hepatocellular Carcinoma and Cholangiocarcinoma—What Do We Already Know. Cancers.

[141] Méndez-Sánchez N., Bugianesi E., Gish R.G., Lammert F., Tilg H., Nguyen M.H., Sarin S.K., Fabrellas N., Zelber-Sagi S., Fan J.-G. (2022). Global multi-stakeholder endorsement of the MAFLD definition. Lancet Gastroenterol. Hepatol..

[142] Di Ciaula A., Bonfrate L., Portincasa P. (2022). The role of microbiota in nonalcoholic fatty liver disease (NAFLD). Eur. J. Clin. Investig..

[143] Di Ciaula A., Bonfrate L., Krawczyk M., Frühbeck G., Portincasa P. (2022). Synergistic and Detrimental Effects of Alcohol Intake on Progression of Liver Steatosis. Int. J. Mol. Sci..

[144] Grattagliano I., Di Ciaula A., Baj J., Molina-Molina E., Shanmugam H., Garruti G., Wang D.Q.-H., Portincasa P. (2021). Protocols for Mitochondria as the Target of Pharmacological Therapy in the Context of Nonalcoholic Fatty Liver Disease (NAFLD). Methods Mol. Biol..

[145] Di Ciaula A., Passarella S., Shanmugam H., Noviello M., Bonfrate L., Wang D.Q.-H., Portincasa P. (2021). Nonalcoholic Fatty Liver Disease (NAFLD). Mitochondria as Players and Targets of Therapies?. Int. J. Mol. Sci..

[146] Di Ciaula A., Calamita G., Shanmugam H., Khalil M., Bonfrate L., Wang D., Baffy G., Portincasa P. (2021). Mitochondria Matter: Systemic Aspects of Nonalcoholic Fatty Liver Disease (NAFLD) and Diagnostic Assessment of Liver Function by Stable Isotope Dynamic Breath Tests. Int. J. Mol. Sci..

[147] Thomas London W., Petrick J.L., McGlynn K.A., Thun M., Linet M.S., Cerhan J.R., Haiman D.S.C.A. (2018). London: Cancer Epidemiology and Prevention. Cancer Epidemiology and Prevention.

[148] Scinicariello F., Sato T., Lee C.S., Hsu H.C., Chan T.S., Tyring S.K. (1992). Detection of human papillomavirus in primary hepatocellular carcinoma—PubMed. Anticancer Res..

[149] Ma T., Su Z., Chen L., Liu S., Zhu N., Wen L., Yuan Y., Lv L., Chen X., Huang J. (2012). Human Papillomavirus Type 18 E6 and E7 Genes Integrate into Human Hepatoma Derived Cell Line Hep G2. PLoS ONE.

[150] Pang R.W.C., Joh J.W., Johnson P.J., Monden M., Pawlik T.M., Poon R.T.P. (2008). Biology of hepatocellular carcinoma. Ann. Surg. Oncol..

[151] Ferber M.J., Montoya D.P., Yu C., Aderca I., McGee A., Thorland E.C., Nagorney D.M., Gostout B.S., Burgart L.J., Boix L. (2003). Integrations of the hepatitis B virus (HBV) and human papillomavirus (HPV) into the human telomerase reverse transcriptase (hTERT) gene in liver and cervical cancers. Oncogene.

[152] Pucułek M., Machlowska J., Wierzbicki R., Baj J., Maciejewski R., Sitarz R. (2018). *Helicobacter pylori* associated factors in the development of gastric cancer with special reference to the early-onset subtype. Oncotarget.

[153] Dudek I., Forma A., Hamerska J., Flieger M., Januszewski J., Cywka T., Kozak J., Baj J. (2022). Helicobacter pylori cytotoxin-associated gene A virulence and its association with the epithelial-mesenchymal transition in gastric cancer. J. Educ. Health Sport.

[154] Kim J., Cho Y.A., Choi W.J., Jeong S.H. (2014). Gene-diet interactions in gastric cancer risk: A systematic review. World J. Gastroenterol..

[155] Moy K.A., Fan Y., Wang R., Gao Y.-T., Yu M.C., Yuan J.-M. (2010). Alcohol and Tobacco Use in Relation to Gastric Cancer: A Prospective Study of Men in Shanghai, China. Cancer Epidemiol. Biomark. Prev..

[156] Duell E.J., Travier N., Lujan-Barroso L., Clavel-Chapelon F., Boutron-Ruault M., Morois S., Palli D., Krogh V., Panico S., Tumino R. (2011). Alcohol consumption and gastric cancer risk in the European Prospective Investigation into Cancer and Nutrition (EPIC) cohort. Am. J. Clin. Nutr..

[157] Ishaq S., Nunn L. (2015). Helicobacter pylori and gastric cancer: A state of the art review. Gastroenterol. Hepatol. Bed Bench.

[158] Iizasa H., Nanbo A., Nishikawa J., Jinushi M., Yoshiyama H. (2012). Epstein-Barr Virus (EBV)-associated Gastric Carcinoma. Viruses.

[159] Baj J., Brzozowska K., Forma A., Maani A., Sitarz E., Portincasa P. (2020). Immunological Aspects of the Tumor Microenvironment and Epithelial-Mesenchymal Transition in Gastric Carcinogenesis. Int. J. Mol. Sci..

[160] Zhang K., Lv D., Li R. (2019). Protein inhibitor of activated STAT1 (PIAS1) inhibits IRF8 activation of Epstein-Barr virus lytic gene expression. Virology.

[161] Snietura M., Waniczek D., Piglowski W., Kopec A., Nowakowska-Zajdel E., Lorenc Z., Muc-Wierzgon M. (2014). Potential role of human papilloma virus in the pathogenesis of gastric cancer. World J. Gastroenterol..

[162] Bae J.-M. (2021). Human papillomavirus infection and gastric cancer risk: A meta-epidemiological review. World J. Virol..

[163] Zeng Z.-M., Luo F.-F., Zou L.-X., He R.-Q., Pan D.-H., Chen X., Xie T.-T., Li Y.-Q., Peng Z.-G., Chen G. (2016). Human papillomavirus as a potential risk factor for gastric cancer: A meta-analysis of 1,917 cases. OncoTargets Ther..

[164] Thanikachalam K., Khan G. (2019). Colorectal Cancer and Nutrition. Nutrients.

[165] Brenner H., Kloor M., Pox C.P. (2014). Colorectal cancer. Lancet.

[166] Gillison M.L., Chaturvedi A.K., Lowy D.R. (2008). HPV prophylactic vaccines and the potential prevention of noncervical cancers in both men and women. Cancer.

[167] Bychkov V.A., Nikitina E.G., Ibragimova M.K., Kaigorodova E.V., Choinzonov E.L., Litviakov N.V. (2016). Comprehensive meta-analytical summary on human papillomavirus association with head and neck cancer. Exp. Oncol..

[168] Kirgan D., Manalo P., Hall M., McGregor B. (1990). Association of Human Papillomavirus and Colon Neoplasms. Arch. Surg..

[169] Bodaghi S., Yamanegi K., Xiao S.-Y., Da Costa M., Palefsky J.M., Zheng Z.-M. (2005). Colorectal Papillomavirus Infection in Patients with Colorectal Cancer. Clin. Cancer Res..

[170] Chen T.H., Huang C.C., Yeh K.T., Chang S.H., Chang S.W., Sung W.W., Cheng Y.W., Lee H. (2012). Human papilloma virus 16 E6 oncoprotein associated with p53 inactivation in colorectal cancer. World J. Gastroenterol..

[171] Pérez L.O., Barbisan G., Ottino A., Pianzola H., Golijow C.D. (2010). Human Papillomavirus DNA and Oncogene Alterations in Colorectal Tumors. Pathol. Oncol. Res..

[172] Ibragimova M.K., Tsyganov M.M., Litviakov N.V. (2018). Human papillomavirus and colorectal cancer. Med. Oncol..

[173] Yavuzer D., Karadayi N., Salepci T., Baloğlu H., Dabak R., Bayramicli O.U. (2010). Investigation of human papillomavirus DNA in colorectal carcinomas and adenomas. Med. Oncol..

[174] Baandrup L., Thomsen L.T., Olesen T.B., Andersen K.K., Norrild B., Kjaer S.K. (2014). The prevalence of human papillomavirus in colorectal adenomas and adenocarcinomas: A systematic review and meta-analysis. Eur. J. Cancer.

[175] Cheng J.Y., Sheu L.F., Lin J.C., Meng C.L. (1995). Detection of human papillomavirus DNA in colorectal adenomas. Arch. Surg..

[176] Burnett-Hartman A.N., Newcomb P.A., Mandelson M.T., Galloway D.A., Madeleine M.M., Wurscher M.A., Carter J.J., Makar K.W., Potter J., Schwartz S.M. (2011). No Evidence for Human Papillomavirus in the Etiology of Colorectal Polyps. Cancer Epidemiol. Biomark. Prev..

[177] Damin D.C., Ziegelmann P.K., Damin A.P. (2013). Human papillomavirus infection and colorectal cancer risk: A meta-analysis. Color. Dis..

[178] Shaw A.R. (2013). Human Papillomavirus Vaccines Six Years After Approval. Annu. Rev. Med..

[179] Cancer Statistics Review, 1975-2014—SEER Statistics. SEER. https://seer.cancer.gov/archive/csr/1975_2014/.

[180] Mirabello L., Clarke M.A., Nelson C.W., Dean M., Wentzensen N., Yeager M., Cullen M., Boland J.F., Schiffman M., Burk R.D. (2018). The Intersection of HPV Epidemiology, Genomics and Mechanistic Studies of HPV-Mediated Carcinogenesis. Viruses.

[181] Celie K.-B., Jackson C., Agrawal S., Dodhia C., Guzman C., Kaufman T., Hellenthal N., Monie D., Monzon J., Oceguera L. (2017). Socioeconomic and gender disparities in anal cancer diagnosis and treatment. Surg. Oncol..

[182] How Many Cancers Are Linked with HPV Each Year?. https://www.cdc.gov/cancer/hpv/statistics/cases.htm.

[183] Li Y., Xu C. (2017). Human Papillomavirus-Related Cancers. Adv. Exp. Med. Biol..

[184] Jin J. (2018). HPV Infection and Cancer. JAMA.

[185] Krzowska-Firych J., Lucas G., Lucas C., Lucas N., Pietrzyk Ł. (2018). An overview of Human Papillomavirus (HPV) as an etiological factor of the anal cancer. J. Infect. Public Health.

[186] de Martel C., Plummer M., Vignat J., Franceschi S. (2017). Worldwide burden of cancer attributable to HPV by site, country and HPV type. Int. J. Cancer.

[187] Shukla S., Bharti A.C., Mahata S., Hussain S., Kumar R., Hedau S., Das B.C. (2009). Infection of human papillomaviruses in cancers of different human organ sites. Indian J. Med. Res..

[188] Senapati R., Senapati N.N., Dwibedi B. (2016). Molecular mechanisms of HPV mediated neoplastic progression. Infect. Agent Cancer.

[189] Chabeda A., Yanez R.J.R., Lamprecht R., Meyers A.E., Rybicki E.P., Hitzeroth I.I. (2018). Therapeutic vaccines for high-risk HPV-associated diseases. Papillomavirus Res..

[190] Castle P.E., Maza M. (2016). Prophylactic HPV vaccination: Past, present, and future. Epidemiol. Infect..

[191] Hildesheim A., Gonzalez P., Kreimer A.R., Wacholder S., Schussler J., Rodriguez A.C., Porras C., Schiffman M., Sidawy M., Schiller J.T. (2016). Impact of human papillomavirus (HPV) 16 and 18 vaccination on prevalent infections and rates of cervical lesions after excisional treatment. Am. J. Obstet. Gynecol..

[192] Gallego L.C., Dominguez A., Parmar M. (2022). Human Papilloma Virus Vaccine. StatPearls.

[193] Jit M., Brisson M., Portnoy A., Hutubessy R. (2014). Cost-effectiveness of female human papillomavirus vaccination in 179 countries: A PRIME modelling study. Lancet Glob. Health.

